# Comparative analysis of oncologic outcomes: kidney-sparing surgery versus radical nephroureterectomy for localized high-risk upper tract urothelial carcinoma

**DOI:** 10.3389/fonc.2026.1753148

**Published:** 2026-02-17

**Authors:** Hongyu Zhang, Qi Tan, Yutao He, Tong Zhang, Yunfeng He

**Affiliations:** Department of Urology, The First Affiliated Hospital of Chongqing Medical University, Chongqing, China

**Keywords:** kidney-sparing surgery, radical nephroureterectomy, renal function, survival, upper tract urothelial carcinoma

## Abstract

**Background:**

The kidney-sparing surgery (KSS) technique for upper tract urothelial carcinoma (UTUC) shows potential, yet its efficacy in treating high-risk UTUC is a topic of debate. This study seeks to assess and compare the impact of KSS versus radical nephroureterectomy (RNU) on renal function and long-term oncological outcomes in patients with localized high-risk UTUC.

**Methods:**

This study retrospectively analyzed 115 patients diagnosed with high-risk UTUC and treated with KSS or RNU in the First Affiliated Hospital of Chongqing Medical University from January 2018 to June 2024. The primary endpoint of this study was overall survival (OS). Secondary endpoints included disease-free survival (DFS), metastasis-free survival (MFS), intravesical recurrence-free survival (IVRFS), and postoperative renal function changes. Additionally, predictors of OS, DFS, MFS, and IVRFS were evaluated through Cox regression models.

**Results:**

There were no significant differences in the 3-year OS (88.1% vs 78.5%), DFS (60.3% vs 53.9%), MFS (82.1% vs 71.9%), and IVRFS (80.2% vs 69.3%) rates between the KSS and RNU groups, which included 29 and 86 patients, respectively. 6 months after intervention, renal function decreased significantly in RNU group, but not in KSS group. In univariate Cox regression analysis, the surgical procedure did not exhibit significant associations with OS, DFS, MFS, or IVRFS. Subsequent multivariable Cox regression analysis revealed that preoperative creatinine levels, pathological stage, and intraoperative bleeding emerged as independent predictors of OS. Moreover, pathological stage was also an independent predictor for DFS and MFS.

**Conclusions:**

KSS exhibits similar oncological efficacy as RNU in managing localized high-risk UTUC, with less damage to patients and benefit of better preservation of renal function. Adverse factors such as elevated preoperative creatinine levels, advanced pathological stage, and increased intraoperative blood loss may correlate with inferior overall survival. Additional prospective research is necessary to confirm the validity of our findings.

## 1 Introduction

Upper tract urothelial carcinoma (UTUC) refers to urothelial carcinoma that originates from the renal pelvis and ureter. This malignancy is relatively rare, comprising only 5% to 10% of all urothelial carcinomas (1). UTUC presents a greater clinical challenge compared to bladder cancer. Chinese patients with UTUC typically present with seemingly good health, yet frequently demonstrate adverse pathological characteristics, such as high-grade pathology and muscular invasion (2). Radical nephroureterectomy (RNU) with cystectomy cuff is the standard treatment for localized high-risk UTUC. However, RNU is associated with a spectrum of postoperative complications and potential renal function impairment in patients. Cisplatin-based postoperative adjuvant therapy is difficult to tolerate in some patients (3), and complications such as chronic kidney disease after RNU may even increase the overall mortality rate (4). Additionally, elderly patients may experience poorer outcomes after RNU compared to their younger patients (5). Kidney-sparing surgery (KSS), including segmental ureterectomy (SU) and endoscopic surgery, may have shorter operating time than RNU, reduce postoperative complications, and protect renal function (6). It has demonstrated satisfactory efficacy in low-risk UTUC patients, but its ability to eliminate tumor cells in high-risk cases is still controversial.

As surgical technology advances and the concept of precision medicine evolves, an increasing number of patients and clinicians are opting for organ-preserving surgical techniques to treat tumors instead of radical surgery under the premise of ensuring the prognosis of oncology. The 2025 version of the European Association of Urology (EAU) guidelines underscores the increasing importance of KSS in managing UTUC. However, there remains a scarcity of robust evidence regarding the oncological outcomes of KSS in high-risk UTUC treatment, necessitating further clinical research to substantiate its efficacy. Apart from the inherent risk factors associated with high-risk UTUC, additional variables influencing the prognosis of patients with high-risk UTUC necessitate further validation. Therefore, we conducted this study at our center to further evaluate the safety and feasibility of KSS in high-risk UTUC patients and provide experience for KSS in high-risk UTUC patients.

## 2 Materials and methods

### 2.1 Study population

**Figure 1:**
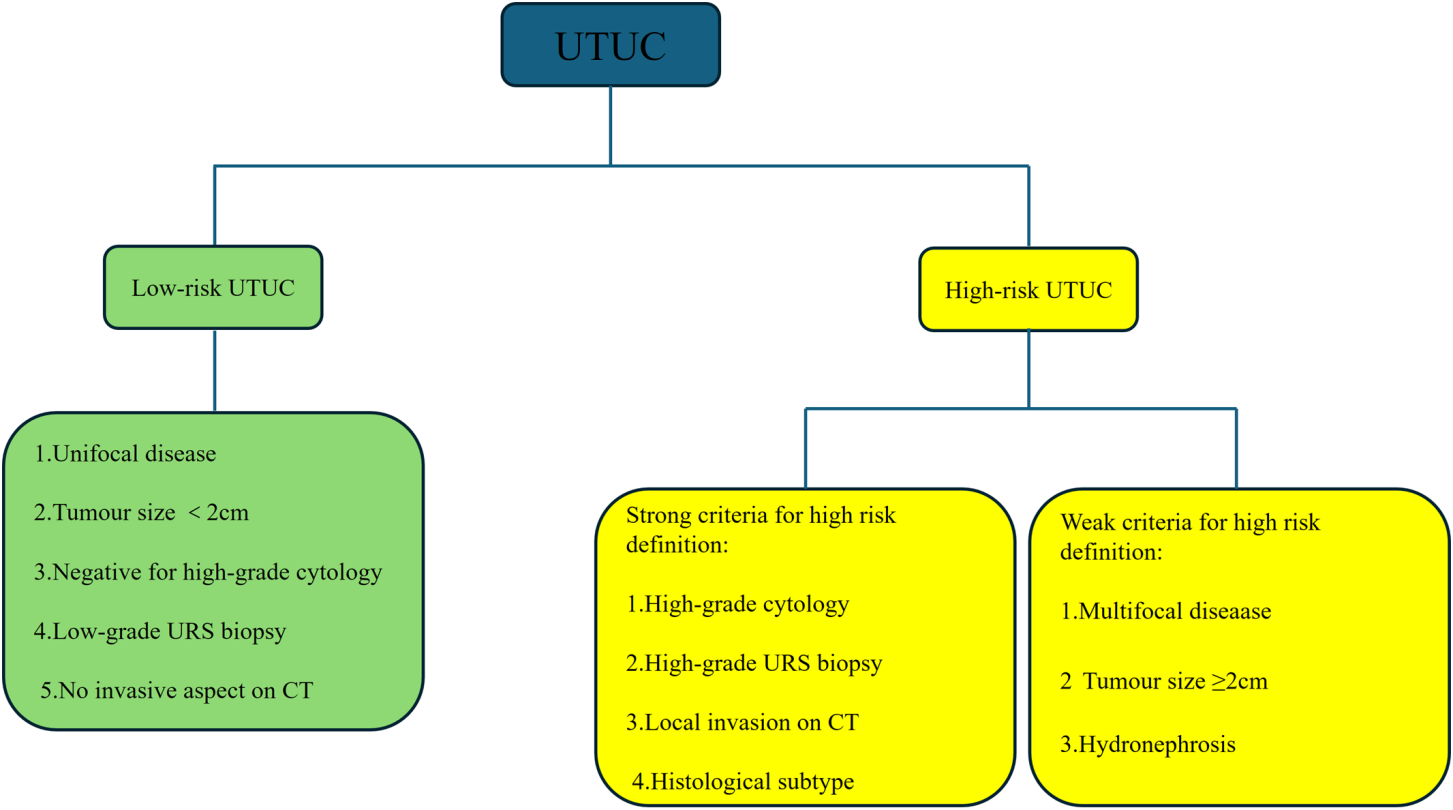
2025 EAU guidelines risk stratification criteria.

This study retrospectively analyzed patients with UTUC who underwent surgical treatment at the First Affiliated Hospital of Chongqing Medical University from January 2018 to June 2024. Inclusion criteria: Patients identified as localized high-risk UTUC according to the latest version of the 2025 European Association of Urology EAU risk stratification criteria. It is worth noting that multifocal, tumor diameter, and hydronephrosis were considered weak criteria for defining high risk in the latest version of risk stratification, and these factors were still listed as high-risk factors in this study, as shown in **Figure 1**. Exclusion criteria: (1) Patients with a follow-up period of less than 6 months or those who underwent neoadjuvant therapy; (2) Patients with distant metastasis or lymph node metastasis; (3) Patients with end-stage renal disease or a solitary kidney; (4) Patients who had previously undergone radical cystectomy for high-grade bladder cancer or radical organ resection for other malignancies; (5) Patients with synchronous bladder cancer or other malignant tumors; (6) Patients with bilateral UTUC.

This study was conducted in accordance with the Helsinki Declaration and approved by the Ethics Committee of Chongqing Medical University Affiliated to Chongqing Medical University. As this study is a retrospective study based on medical records, the requirement to obtain signed informed consent was waived.

### 2.2 Data collection

Patients were categorized into two groups according to the treatment they received: the RNU group and the KSS group. Data concerning the study participants, encompassing baseline characteristics, tumor-related indicators, surgery-related indicators, adjuvant therapy, and surgical outcomes, were extracted from the electronic medical record system for a thorough analysis. All study subjects were followed up for at least 6 months postoperatively. Collect postoperative follow-up data through electronic medical record system and telephone interviews. Typically, patients underwent follow-up every 3–4 months within the first 2 years after surgery, every 6 months from the third to the fifth year, and annually thereafter. Each patient who received KSS was informed that the risk of recurrence might be higher than with radical surgery, and that close, active follow-up, including regular cystoscopic or ureteroscopic surveillance, was an essential component of successful KSS management, with the understanding that repeat procedures might be necessary. The primary endpoint of this study was overall survival (OS). Secondary endpoints included disease-free survival (DFS), metastasis-free survival (MFS), intravesical recurrence-free survival (IVRFS), and postoperative renal function changes.

### 2.3 Surgical procedures

The clinicians provided detailed explanations of the two treatment options (RNU and KSS) to all participants using standardized terminology. Patients were educated that RNU is currently the established treatment for high-risk UTUC and were given autonomy to select their preferred treatment approach. The RNU procedure was performed according to the standard surgical protocol for radical nephroureterectomy with bladder cuff excision. The KSS surgical interventions comprised SU and endoscopic procedures, with the majority of enrolled patients undergoing SU. The SU process conformed to the customary procedure, wherein post-resection of the afflicted ureteral segment, the choice of end-to-end anastomosis of the ureter, ureteral reimplantation into the bladder, or anastomosis between the ureter and renal pelvis was based on the location of the lesion. Endoscopic treatment primarily involved accessing the affected ureter via an ureteroscope through the urethra and employing laser ablation or electrocautery with a resection loop for tumor removal.

### 2.4 Statistical analysis

Statistical analyses were conducted using SPSS 21.0, with normally distributed continuous variables expressed as mean ± SD (independent-samples t-test for intergroup comparisons; paired t-test for preoperative-postoperative comparisons) and skewed data as median (IQR) (Mann-Whitney U test for intergroup; Wilcoxon signed-rank test for paired comparisons). Categorical data were analyzed by Pearson χ2, Yates-corrected χ2, or Fisher’s exact test based on theoretical frequency. Kaplan-Meier survival curves were generated to depict postoperative survival rates, and Log-rank tests were used for intergroup comparisons. Univariate and multivariate Cox proportional hazards regression models were employed to identify prognostic factors in high-risk ureteral carcinoma. A two-sided P-value < 0.05 was considered statistically significant.

## 3 Results

### 3.1 Baseline characteristics

**Figure 2:**
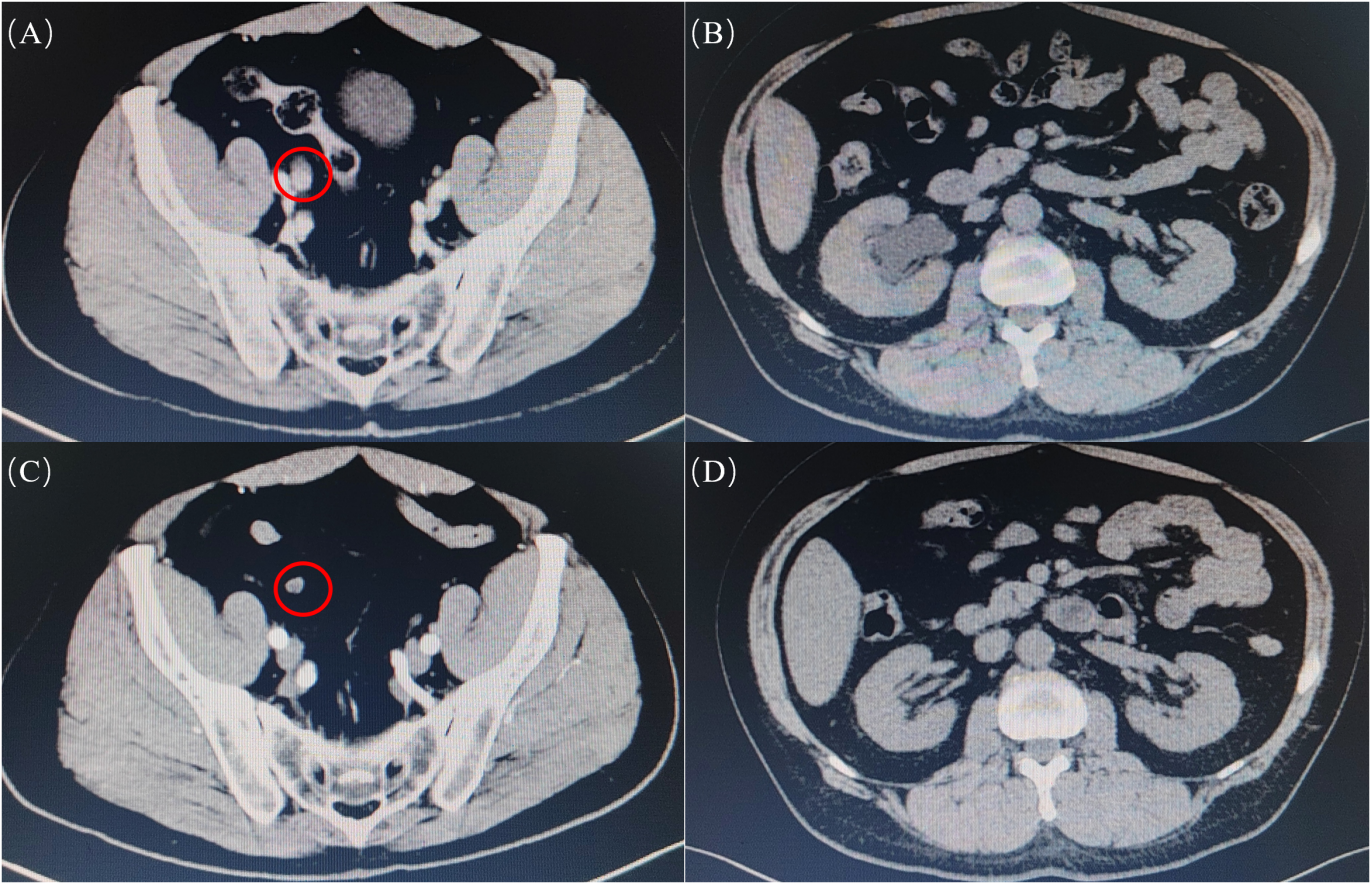
Preoperative and postoperative CT images of SU patients were received. **(A)** Preoperative ureteral tumor site of the patient; **(B)** Preoperative hydronephrosis; **(C)** No recurrence was found in the operation area of the same patient 19 months after operation; **(D)** No hydronephrosis was found in the same patient 19 months after operation.

A total of 115 patients with high-risk UTUC were collected and included in this study, including 86 patients undergoing RNU and 29 patients who underwent KSS. Among them, the KSS group included 23 patients undergoing SU and 6 patients undergoing endoscopic surgery. The imaging results before and after treatment were shown in **Figure 2**.

General clinical data and tumor characteristics of the two groups are shown in **Table 1**. There were no significant differences in age, BMI, gender, smoking history, hematuria, lumbago, hydronephrosis, preoperative creatinine and preoperative eGFR between KSS group and RNU group (P>0.05). The KSS group exhibited significantly higher prevalence rates of diabetes (27.6% vs. 9.3%, P = 0.032) and coronary heart disease (20.7% vs. 4.7%, P = 0.023) compared to the RNU group. Tumor laterality differed significantly between groups (right-sided predominance in KSS vs left-sided in RNU, P = 0.036). No significant difference existed in pathological types (P = 0.128), though notably, 3 patients had histological subtypes:the KSS group included 1 case of squamous cell carcinoma and 1 case of large cell neuroendocrine carcinoma, while the RNU group had 1 case of poorly differentiated adenocarcinoma.

**Table 1:** Comparison of general clinical data of patients.

Characteristics		KSS(n=29)	RNU(n=86)	t/χ²/Z	P value
Age (years)		68.31 ± 10.13	67.65 ± 8.09	0.355	0.723
BMI(kg/m^2^)		24.28 ± 3.32	23.59 ± 2.71	1.128	0.262
Gender, n (%)	Male	18(62.1%)	46(53.5%)	0.647	0.421
	Female	11(37.9%)	40(46.5%)		
Smoking history, n (%)		10(34.5%)	28(32.6%)	0.036	0.849
Hypertension, n (%)		15(51.7%)	35(40.7%)	1.073	0.300
Diabetes, n (%)		8(27.6%)	8(9.3%)	4.623	**0.032**
Coronary heart disease, n (%)		6(20.7%)	4(4.7%)	5.151	**0.023**
Hematuria, n (%)		22(75.9%)	59(68.6%)	0.549	0.459
Lumbago, n (%)		9(31.0%)	31(36.0%)	0.240	0.624
Hydronephrosis, n (%)		23(79.3%)	79(91.9%)	2.270	0.132
Preoperative creatinine (µmol/l)		93(79.50~131.50)	102(78.75~125)	-0.441	0.659
Preoperative eGFR (ml/min/1.73 m^2^)		68.91 ± 26.53	63.36 ± 20.42	1.029	0.310
Tumor					
Laterality, n (%)	Left	9(31.0%)	46(53.5%)	4.382	**0.036**
	Right	20(69.0%)	40(46.5%)		
Single or multifocal, n (%)	Single	28(96.6%)	77(89.5%)	0.606	0.436
	Multifocal	1(3.4%)	9(10.5%)		
Location, n (%)	Renal pelvis and upper ureter	1(3.4%)	13(15.1%)	4.157	0.114
	middle and lower ureter	27(93.1%)	64(74.4%)		
	Both	1(3.4%)	9(10.5%)		
Diameter(cm)		2.30(1.75~3.00)	2.48(1.70~3.01)	-0.355	0.723
Pathological type, n (%)	Low grade UC	6(20.7%)	12(14.0%)	3.756	0.128
	High grade UC	21(72.4%)	73(84.9%)		
	Tumors with histological subtype	2(6.9%)	1(1.2%)		
Clinical T stage, n (%)	<cT1	3(10.3%)	6(7.0%)	3.167	0.527
	cT1	14(48.3%)	31(36.0%)		
	cT2	7(24.1%)	21(24.4%)		
	cT3	4(13.8%)	24(27.9%)		
	cT4	1(3.4%)	4(4.7%)		
Pathological T stage, n (%)	<pT1	5(17.2%)	11(12.8%)	5.226	0.236
	pT1	13(44.8%)	23(26.7%)		
	pT2	6(20.7%)	29(33.7%)		
	pT3	4(13.8%)	21(24.4%)		
	pT4	1(3.4%)	2(2.3%)		

Bold values indicate statistical significance, defined as P<0.05.

### 3.2 Perioperative indicators and postoperative treatment

The mean operative time of the KSS group was 140.07 ± 79.51 min, and that of the RNU group was 198.02 ± 50.82 min, with statistical significance (P<0.001). The estimated median intraoperative blood loss of the KSS group and RNU group was 50ml (IQR: 40ml to 150ml) and 200ml (IQR: 100ml to 300ml), respectively, with statistical significance (P<0.001).The operation time and intraoperative blood loss of the KSS group were lower than those of the RNU group, but there was no significant difference in postoperative hospital stay between the two groups (P>0.05). Regarding complications, the radical operation group experienced 13 cases of Clavien II complications and 3 cases of complications exceeding Grade II, whereas the kidney preservation group had 2 cases of Clavien II complications and no complications exceeding Clavien II.

Postoperative adjuvant therapy was administered to 19 KSS patients versus 69 RNU patients. Adjuvant therapy included bladder instillation, immunotherapy, systemic chemotherapy and radiotherapy, with 7 KSS patients and 24 RNU patients received combination regimens (≥2 treatment modalities). No intergroup difference was observed in adjuvant therapy utilization (P>0.05). Perioperative and postoperative treatment indicators of the two groups are shown in **Table 2**.

**Table 2:** Comparison of perioperative related indicators and postoperative treatment metrics in patients.

Characteristics		KSS(n=29)	RNU(n=86)	t/χ²/Z	P value
Operation time (min)		140.07 ± 79.51	198.02 ± 50.82	-3.680	**<0.001**
Intraoperative blood loss(ml)		50(40~150)	200(100~300)	-4.761	**<0.001**
Postoperative hospital stay(days)		6.00(3.00~8.50)	5.00(4.00~7.00)	-0.276	0.783
Complication grade, n (%)	Clavien II	2(6.9%)	13(15.1%)	1.764	0.385
	> Clavien II	0(0.0%)	3(3.5%)		
Adjuvant therapy, n (%)		19(65.5%)	69(80.2%)	2.614	0.106
Combination regimens, n (%)		7(24.1%)	24(27.9%)	0.156	0.692
Specific adjuvant treatment regimens, n (%)	Bladder instillation	14(48.3%)	53(61.6%)	1.590	0.207
	Immunotherapy	6(20.7%)	14(16.3%)	0.294	0.588
	Systemic chemotherapy	7(24.1%)	30(34.9%)	1.148	0.284
	Radiotherapy	1(3.4%)	0(0.0%)		0.252

Bold values indicate statistical significance, defined as P<0.05.

### 3.3 Change in renal function

It can be seen from the above that there was no statistically significant difference in preoperative creatinine and preoperative eGFR between the two groups. However, at the 6-month postoperative follow-up, the KSS group demonstrated significantly lower serum creatinine levels (P <0.001) and higher eGFR values (P <0.001) compared to the RNU group. Further analysis of the relative changes from baseline showed a distinct functional advantage for KSS. The percentage change in creatinine was -8.6% (IQR: -18.9% to 6.4%) in the KSS group, compared to 15.4% (IQR: 2.6% to 39.9%) in the RNU group (P < 0.001). Similarly, the change rate in eGFR was 13.0% (IQR: -8.7% to 23.9%) in the KSS group, whereas it was -16.2% (IQR: -31.8% to 4.5%) in the RNU group (P < 0.001).The postoperative renal function changes of the two groups of patients are detailed in **Table 3**.

**Table 3:** Postoperative renal function indexes of patients.

Characteristics	KSS(n=29)	RNU(n=86)	t/χ²/Z	P value
6-month postoperative creatinine(µmol/l)	89(77~107)	110(100~135)	-4.390	**<0.001**
6-month postoperative eGFR (ml/min/1.73 m^2^)	71.79 ± 19.36	52.62 ± 15.03	5.509	**<0.001**
rate of change in creatinine (%)	-8.6(-18.9~6.4)	15.4(-2.6~39.9)	-3.919	**<0.001**
rate of change in eGFR (%)	13.0(-8.7~23.9)	-16.21(-31.8~4.5)	-3.845	**<0.001**

Bold values indicate statistical significance, defined as P<0.05.

**Figure 3:**
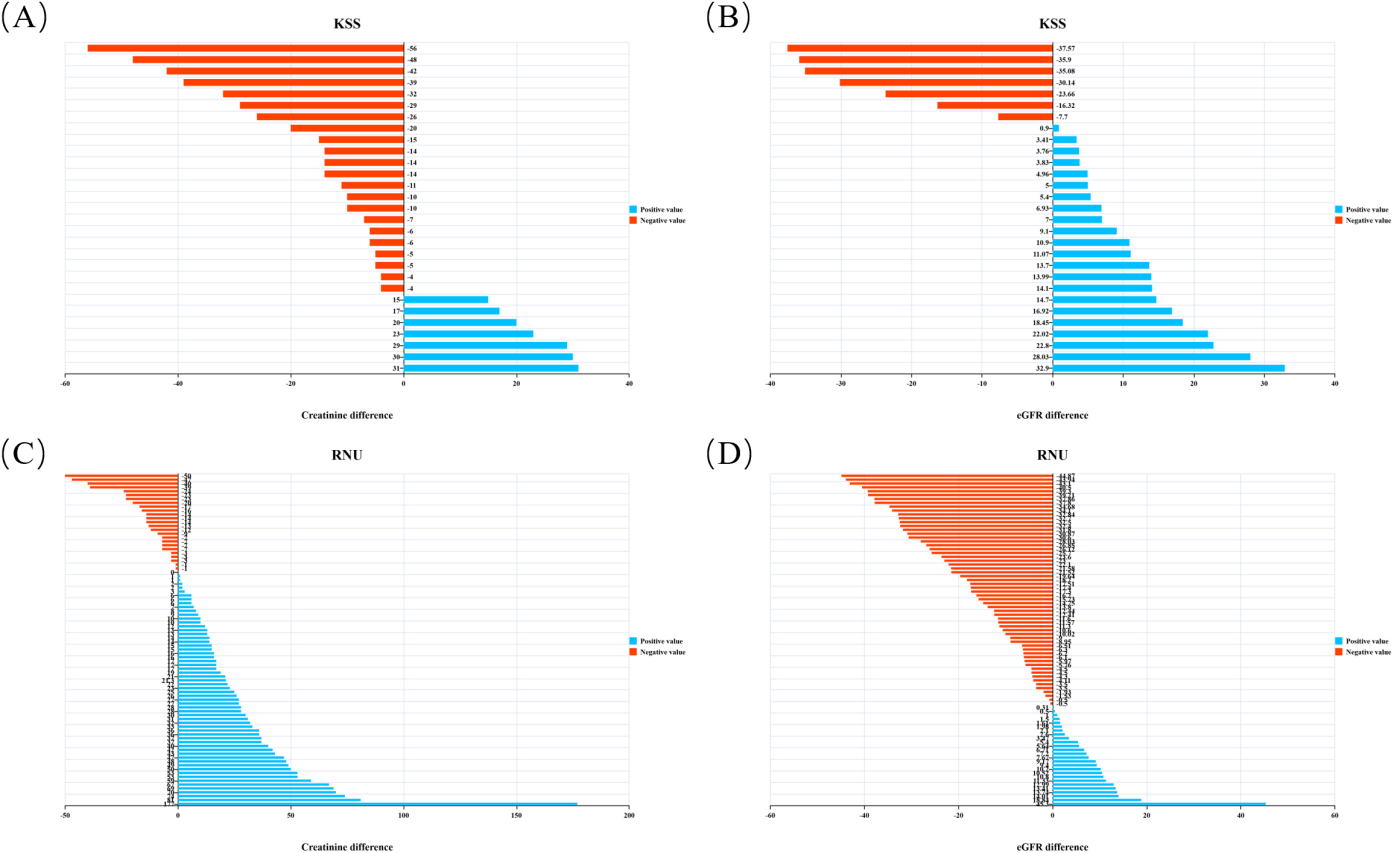
**(A)** Difference between creatinine and preoperative creatinine at 6 months after operation in KSS group; **(B)** Difference between eGFR and preoperative eGFR at 6 months after operation in KSS group; **(C)** Difference between creatinine and preoperative creatinine at 6 months after operation in RNU group; **(D)** Difference between eGFR and preoperative eGFR at 6 months after operation in RNU group. Blue denotes a relative increase and red a relative decrease in comparison to baseline.

At the 6-month postoperative follow-up, the average eGFR in the RNU group decreased by 10.75 ± 17.59 ml/min/1.73 m^2^ compared with before surgery (P<0.001), mean creatinine increased by 16.25 ± 32.41 µmol/L (P<0.001). In contrast, the KSS group demonstrated a non-significant mean eGFR improvement of 2.88 ± 19.20 ml/min/1.73 m² (P = 0.426) and a borderline significant mean creatinine decrease of 8.69 ± 23.11 µmol/L (P = 0.053). **Figure 3** demonstrates changes in renal function before and after surgery.

### 3.4 Survival outcomes

The median follow-up time for patients was 33.0 months (IQR:19.0–45.0 months). There were 17 deaths in RNU group and 5 deaths in KSS group. In the RNU group, 35 cases experienced recurrence, of which 22 cases had bladder recurrence; In the KSS group, there were 12 instances of recurrence, comprising 6 cases of bladder recurrence and 2 cases of ipsilateral ureter recurrence. One of the patients with ipsilateral ureter recurrence received SU treatment 9 months after the initial endoscopic procedure, while the other received systemic adjuvant therapy.

**Figure 4:**
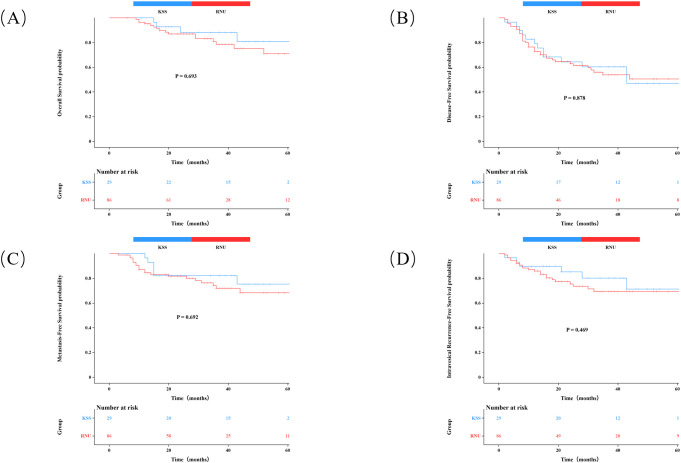
**(A)** Overall survival rate (OS); **(B)** Disease-free survival (DFS); **(C)** Metastasis-free survival (MFS); **(D)** Intravesical recurrence-free survival.

Kaplan-Meier survival curves were used to analyze OS, DFS, MFS, and IVRFS of patients in different treatment groups, and Log-rank test was performed. MFS was defined as the interval from treatment initiation to first occurrence of distant metastasis or death. IVRFS was defined as the duration from surgical treatment to bladder tumor recurrence (excluding recurrences at ureteral sites). The 3-year OS rates were 78.5% for RNU and 88.1% for KSS (P = 0.693). Corresponding 3-year rates for DFS, MFS, and IVRFS were 53.9% vs. 60.3% (P = 0.878), 71.9% vs. 82.1% (P = 0.692), and 69.3% vs. 80.2% (P = 0.469), respectively. None of the intergroup differences were statistically significant. As shown in **Figure 4**.

### 3.5 Analysis of prognostic factors

Given that all cases in this study involved localized high-risk UTUC, variables confirmatory of high-risk UTUC were excluded from regression analyses. Univariate analysis demonstrated no significant association between surgical method and OS, DFS, MFS, or IVRFS in high-risk UTUC. Multivariate Cox regression analysis was conducted on statistically significant factors in univariate analysis. The results showed that preoperative creatinine, pathological staging, and intraoperative blood loss were independent predictive factors for OS (all P < 0.05), while pathological staging was also an independent predictive factor for DFS and MFS (all P < 0.05). No significant independent predictors were identified for IVRFS, as shown in **Table 4**.

**Table 4:** Univariate and multivariate Cox regression analysis of prognostic factors in high-risk UTUC.

Characteristics		OS			DFS			MFS			IVRFS		
		HR	95% CI	P	HR	95% CI	P	HR	95% CI	P	HR	95% CI	P
Univariate Cox regression analysis													
Surgical method	RNU	reference			reference			reference			reference		
	KSS	0.816	0.297-2.241	0.694	0.952	0.504-1.797	0.879	0.842	0.357-1.985	0.694	0.718	0.291-1.773	0.473
Age		1.007	0.961-1.056	0.76	0.994	0.963-1.027	0.734	1.007	0.965-1.051	0.749	0.985	0.945-1.026	0.458
BMI		0.958	0.823-1.116	0.585	0.983	0.892-1.083	0.723	0.945	0.829-1.078	0.401	0.985	0.866-1.120	0.813
Gender	Male	reference			reference			reference			reference		
	Female	0.54	0.220-1.327	0.176	0.627	0.376-1.202	0.180	0.552	0.249-1.223	0.143	0.761	0.356-1.625	0.480
Smoking history	No	reference			reference			reference			reference		
	Yes	2.531	1.092-5.865	**0.030**	0.91	0.501-1.654	0.758	1.329	0.622-2.838	0.463	0.778	0.342-1.766	0.548
Hypertension	No	reference			reference			reference			reference		
	Yes	2.037	0.868-4.783	0.102	1.408	0.803-2.468	0.232	1.656	0.787-3.482	0.184	1.014	0.479-2.146	0.97
Diabetes	No	reference			reference			reference			reference		
	Yes	0.917	0.271-3.105	0.889	0.872	0.371-2.052	0.754	0.948	0.328-2.737	0.921	1.107	0.383-3.202	0.851
Coronary heart disease	No	reference			reference			reference			reference		
	Yes	0.305	0.041-2.292	0.249	1.034	0.409-2.613	0.943	0.558	0.131-2.368	0.429	1.608	0.557-4.641	0.38
Hematuria	No	reference			reference			reference			reference		
	Yes	1.668	0.612-4.543	0.317	1.753	0.894-3.438	0.102	1.743	0.705-4.310	0.229	1.738	0.703-4.295	0.231
Lumbago	No	reference						reference			reference		
	Yes	1.556	0.664-3.645	0.309	1.441	0.809-2.569	0.215	1.392	0.651-2.978	0.394	1.387	0.648-2.967	0.4
Preoperative creatinine		1.011	1.002-1.020	**0.015**	1.006	0.999-1.012	0.09	1.005	0.997-1.014	0.227	1.009	1.001-1.017	**0.03**
Preoperative eGFR		0.982	0.964-1.001	0.068	0.989	0.977-1.001	0.072	0.989	0.973-1.006	0.197	0.983	0.967-0.999	**0.04**
Tumor													
Laterality	Left	reference			reference			reference			reference		
	Right	1.031	0.444-2.394	0.943	0.969	0.553-1.696	0.911	0.857	0.407-1.805	0.684	0.782	0.372-1.644	0.516
Location	Renal pelvis and upper ureter	reference			reference			reference			reference		
	middle and lower ureter	0.769	0.176-3.352	0.726	0.549	0.196-1.536	0.253	0.895	0.266-3.009	0.858	0.216	0.029-1.595	0.133
	Both	2.787	0.918-8.464	0.071	1.15	0.453-2.919	0.769	2.041	0.698-5.969	0.193	0.677	0.160-2.861	0.596
Pathological T	≤pT2	reference			reference			reference			reference		
	>pT2	3.625	1.557-8.436	**0.003**	2.211	1.223-3.997	**0.009**	4.187	1.989-8.821	**<0.001**	0.775	0.294-2.041	0.606
Operation time		1.002	0.996-1.009	0.528	1.001	0.996-1.005	0.779	1.002	0.996-1.007	0.562	1	0.994-1.006	1.000
Intraoperative blood loss		1.001	1.000-1.002	**0.010**	1	1.000-1.001	0.286	1.001	1.000-1.002	**0.035**	0.999	0.997-1.002	0.631
Adjuvant therapy	No	reference			reference			reference			reference		
	Yes	0.753	0.293-1.938	0.557	1.674	0.784-3.575	0.183	1.037	0.418-2.568	0.938	2.033	0.705-5.864	0.189
Multivariate Cox regression analysis													
Smoking history	No	reference											
	Yes	1.836	0.774-4.356	0.168									
Preoperative creatinine		1.011	1.000-1.021	**0.040**	1.005	0.998-1.012	0.135				1.004	0.986-1.022	0.679
Preoperative eGFR											0.989	0.956-1.023	0.521
Pathological T	≤pT2	reference			reference			reference					
	>pT2	3.232	1.356-7.703	**0.008**	2.131	1.178-3.857	**0.012**	4.051	1.923-8.531	**<0.001**			
Intraoperative blood loss		1.001	1.000-1.002	**0.008**				1.001	1.000-1.002	0.070			

Bold values indicate statistical significance, defined as P<0.05.

## 4 Discussion

KSS encompasses surgical approaches ranging from SU to endoscopic treatment (7). With the development of surgical technology and the deepening of the concept of precision medicine treatment, organ-preserving minimally invasive surgery has become the dominant paradigm in contemporary oncology therapeutic strategy. RNU combined with bladder sleeve resection is still the gold standard surgical treatment for high-risk renal pelvic and ureteral cancer (1). Even in cases of metastatic UTUC (mUTUC), combining nephroureterectomy with systemic therapy has been shown to improve survival in patients with early local disease (T1–T2) compared to systemic treatment alone (8). However, RNU may result in a range of complications, and the risk of perioperative complications is similar between open RNU and laparoscopic RNU (9, 10). One study showed that 14.1% of patients had postoperative complications after RNU, and 3.7% of them were classified as severe complications (11). Compared with RNU, KSS preserves the ipsilateral kidney by segmental ureterectomy or endoscopic direct tumor elimination. In patients with low-risk UTUC, a large number of studies have shown that there is no significant difference in the oncological outcomes between KSS and RNU (12–14). In recent years, there have been more studies on KSS for high-risk UTUC.A retrospective study of 252 participants showed that KSS was also a feasible choice for patients with UTUC, not only in low-risk patients, but also in high-risk patients with normal contralateral kidneys (15). In our study, the operation time and intraoperative blood loss in the KSS group were significantly lower than those in the RNU group. In terms of complications, although there was no statistically significant difference between the two groups, there were no complications higher than Clavien II in the KSS group, which may be partly why patients with high quality of life expectations prefer to receive KSS treatment.

When possible, one of the reasons why people prefer KSS is its protection of renal function. Since RNU requires removal of the entire ureter segment and ipsilateral kidney, it carries a consequential risk of postoperative renal functional impairment (16, 17). Studies report that even patients with normal preoperative, renal function decreases by one-third after RNU and does not show evidence of recovery over time (18). Conversely, research on KSS demonstrates significantly superior eGFR levels and eGFR preservation rates in patients undergoing segmental ureterectomy compared to RNU cohorts at both 1-month and 1-year postoperative intervals (19). Another study showed that while KSS exhibits significantly better eGFR outcomes at the 3-month postoperative mark versus RNU, no statistically significant differences were observed at 2 years (P = 0.081) or 5 years (P = 0.304) post-surgery (20). The study by Huang Z et al. showed a significant increase in mean postoperative eGFR of 4.60 ml/min/1.73 m^2^ in SU group (21). In this study, the mean preoperative eGFR values were 68.91 ml/min/1.73 m² in the KSS group versus 63.36 ml/min/1.73 m² in the RNU group. At 6 months postoperatively, these values measured 71.79 ml/min/1.73 m² and 52.62 ml/min/1.73 m², respectively (p<0.001), while the change rate of eGFR in KSS group and RNU group were 13.0% and -16.2%, respectively (p<0.001). Concomitantly, there were significant differences in postoperative creatinine levels and rates of change between the two groups, reflecting the advantages of KSS in preserving renal function. According to our clinical experience, KSS may protect renal function because most high-risk UTUCs have hydronephrosis, and KSS surgery relieves tumor obstruction while preserving the kidney (**Figure 2**).

Under the condition of ensuring the prognosis of oncology, comprehensive treatment based on KSS is a reasonable choice for the treatment of upper urinary tract urothelial carcinoma. Adjuvant therapy includes bladder instillation, immunotherapy, systemic chemotherapy and radiotherapy. Adjuvant therapy can improve the prognosis and prolong the life of patients (22–26). In our study, the two groups of patients received these postoperative adjuvant therapies, and there was no statistically significant difference between the two groups of patients receiving various adjuvant therapies. The prognosis of patients in RNU group and KSS group is the focus of this study. A study evaluated and analyzed the recurrence-free survival, overall survival, and cancer-specific survival of SU and RNU in high-risk ureteral carcinoma, respectively, and found no significant difference (21). Chen Y et al. analyzed endoscopic cryoablation versus radical nephroureterectomy in high-risk UTUC patients and showed no significant difference in 2-year OS (82% vs 84%), PFS (73% vs 71%), and IVRFS (81% vs 83%) rates (27). These are similar to our results. In our study focusing on high-risk UTUC, where no statistically significant differences existed between the KSS and RNU groups regarding factors such as tumor stage or adjuvant therapy, the 3-year overall survival rate was 88.1% and78.5%, 3-year DFS was 60.3% and 53.9%, 3-year MFS was 82.1% and 71.9%, 3-year IVRFS was 80.2% and 69.3%, respectively. Notably, all intergroup differences lacked statistical significance (p>0.05). COX regression analysis also demonstrated that surgical method was not associated with oncological prognosis.

In order to explore more factors that may affect the prognosis of patients with UTUC, in addition to the high risk factors for the diagnosis of high-risk UTUC, multivariate COX regression analysis showed that tumor pathological stage were independent predictors of OS, DFS and MFS. The new version of the guidelines pointed out that the main prognostic factor of UTUC was tumor pathological stage (1), which was the same as our research results. According to our clinical experience, the pathological stage of tumor and other high-risk factors can often affect the difficulty of operation, resulting in the difference of intraoperative blood loss. Therefore, intraoperative blood loss may have the potential value of indirectly evaluating the prognosis of oncology and can be used as one of the reference indicators of clinical prognosis. In addition, creatinine was considered an independent predictor of OS (P = 0.04) but eGFR was not, suggesting that creatinine values may be more sensitive than eGFR in some settings. Elevated creatinine is often associated with hydronephrosis, which is a weak risk factor for UTUC, and multiple studies have shown that it is associated with poor prognosis for UTUC (28, 29). Preoperative creatinine has prognostic value for OS, but not for tumor specific endpoints (DFS, MFS, IVRFS). In order to clarify this difference, preoperative creatinine may affect OS by increasing the burden of competitive risk comorbidity. However, tumor recurrence and metastasis are more directly dependent on the invasiveness of the tumor itself, such as its pathological stage. In summary, patients presenting with elevated preoperative creatinine, advanced pathological stage, and greater intraoperative blood loss may have a poorer prognosis. This suggests that clinicians should adopt the most aggressive and individualized strategies regarding adjuvant therapy and follow-up planning for such patients. Although this study is retrospective and observational, these markers provide a valuable direction for developing a comprehensive prognostic model for UTUC in the future.

Admittedly, due to the low incidence of UTUC, the sample size we collected was limited. And for ethical reasons, randomization or blinding was not possible, so this study was a non-randomized retrospective study. Although most baseline data were not significantly different between the two groups, there were unidentified confounding variables. Whether KSS is applicable to all high-risk UTUC is still controversial. In the future, well-designed multicenter, randomized controlled or prospective large-sample studies are still needed to further verify the clinical application value of KSS in high-risk UTUC patients, especially for high-risk UTUC defined according to EAU strong standards.

## 5 Conclusions

In conclusion, this study shows that the oncological prognosis of KSS is comparable to RNU, and that KSS has a significant renal function protection advantage at short-and medium-term follow-up, providing evidence for the feasibility of renal sparing therapy in high-risk UTUC patients. In addition, we found factors that may affect the prognosis of patients (including pathological stage, preoperative creatinine, intraoperative blood loss) through analysis, which will provide an important basis for us to predict the disease development more accurately and for personalized adjustment of clinical treatment plan. Despite these insights, the findings must be interpreted considering the study’s limitations, including its single center retrospective design and the potential selection bias and insufficient statistical power that may arise from small sample sizes (especially in the KSS group), which may limit the generalizability of extrapolation of research results. There is a pressing need for prospective, multi-institutional studies with larger cohorts, longer follow-up, and standardized patient selection criteria to definitively validate the role of KSS in this high-risk setting.

## Data Availability

The original contributions presented in the study are included in the article/supplementary material. Further inquiries can be directed to the corresponding author.
